# Prevalence of Diabetes Mellitus Among People Living With HIV and Receiving Antiretroviral Therapy at Muhimbili National Hospital in Tanzania

**DOI:** 10.7759/cureus.96800

**Published:** 2025-11-13

**Authors:** Elvis V Msuya, Edina Kalinga, Lucy Sangu, Elisha F Osati, Raphael Z Sangeda

**Affiliations:** 1 Department of Pharmaceutical Microbiology, Muhimbili University of Health and Allied Sciences, Dar es Salaam, TZA; 2 Department of Internal Medicine, Muhimbili National Hospital, Dar es Salaam, TZA

**Keywords:** antiretroviral therapy, diabetes mellitus, hiv, integrated care, non-communicable diseases, prevalence, risk factors, tanzania

## Abstract

Background

With the widespread use of antiretroviral therapy (ART), people living with HIV (PLWHIV) are experiencing an extended life expectancy and a rising burden of non-communicable diseases, including diabetes mellitus. The interaction between HIV infection, long-term ART exposure, and metabolic alterations remains poorly understood, particularly in sub-Saharan Africa. This study aimed to determine the prevalence of diabetes mellitus and identify associated risk factors among PLWHIV receiving ART in Tanzania.

Methodology

A cross-sectional study was conducted among 409 PLWHIV attending the Muhimbili National Hospital HIV Care and Treatment Clinic in Dar es Salaam, Tanzania. Data on sociodemographic, clinical, and laboratory characteristics were collected using a structured questionnaire and patient medical records. Variables included age, gender, CD4 count, body mass index, ART regimen and duration, and family history of diabetes. Diabetes mellitus was defined according to the WHO criteria as fasting plasma glucose ≥7.0 mmol/L or documented use of glucose-lowering medication. Logistic regression analysis was used to identify factors independently associated with diabetes, and statistical significance was set at a p-value <0.05.

Results

The mean age of the participants was 44.7 years, and 65.3% (n = 267) were female. The prevalence of diabetes mellitus was 3.7% (n = 15). Independent predictors of diabetes included older age (adjusted odds ratio (AOR) = 1.14; 95% confidence interval (CI) = 1.05-1.25; p = 0.003), absence of a family history of diabetes (AOR = 0.07; 95% CI = 0.02-0.27; p < 0.001), and CD4 count <200 cells/µL (AOR = 0.14; 95% CI = 0.02-0.88; p = 0.036). The majority of participants (89.4%) had CD4 count ≥200 cells/µL, and 98.3% achieved viral load suppression.

Conclusions

Diabetes mellitus was observed in 3.7% of PLHIV receiving ART in this clinic cohort. These findings support the need to maintain routine opportunistic blood glucose screening and reinforce metabolic health counseling within HIV care. However, we cannot infer excess risk compared to the general Tanzanian population because no age-standardized comparison was performed, and diagnostic ascertainment was limited to fasting plasma glucose.

## Introduction

The intersection of HIV and non-communicable diseases (NCDs), such as diabetes mellitus, has become an important global health concern, especially in sub-Saharan Africa (SSA), where the HIV burden remains high. The widespread rollout of antiretroviral therapy (ART) has transformed HIV from a fatal infection into a chronic, manageable condition, substantially prolonging survival among people living with HIV (PLWHIV) [1]. For example, ART coverage among PLWHIV increased from 47% in 2010 to 62% in 2018 globally, with countries such as Zimbabwe reporting 86.8% ART coverage and a 45% decline in AIDS-related deaths during the same period [2]. However, as life expectancy improves, HIV programs face a growing challenge in managing long-term metabolic and cardiovascular complications [3].

Globally, the incidence of diabetes is rising disproportionately in low- and middle-income countries, where healthcare systems are often under-resourced to handle the dual epidemics of infectious and chronic diseases. Interactions between HIV infection, ART exposure, and metabolic disorders remain complex. Specific ART regimens, particularly those including protease inhibitors, have been linked to insulin resistance and impaired glucose tolerance, with diabetes prevalence ranging from 3% to 6% among PLWHIV in prior studies [4,5]. These metabolic disturbances may result from ART-induced mitochondrial dysfunction, chronic inflammation, or immune reconstitution.

Emerging evidence from Tanzania and South Africa shows that PLWHIV on ART are at a heightened risk of NCDs, including hypertension, dyslipidemia, and abnormal glucose metabolism [6,7]. In addition, a study involving over 1,300 HIV patients in Dar es Salaam reported a considerable burden of NCDs and mental health conditions, particularly among older and overweight patients [8,9]. This highlights the urgent need for integrated health services that encompass both HIV management and NCD prevention [4,10].

Despite this growing recognition, limited data exist on the burden and determinants of diabetes among PLWHIV in Tanzania. Understanding how demographic, clinical, and treatment-related factors contribute to the risk of diabetes in this population is crucial for developing integrated care models.

Therefore, this study aimed to determine the prevalence of diabetes mellitus and identify associated risk factors among PLWHIV attending the Muhimbili National Hospital (MNH) in Dar es Salaam, Tanzania.

## Materials and methods

Study design and setting

This cross-sectional study was conducted between January and June 2024 at the HIV Care and Treatment Clinic of the MNH, the largest tertiary national referral and teaching hospital in Tanzania. The clinic provides comprehensive HIV services to more than 10,000 patients annually and serves as a national model for integrated HIV and NCD care.

Study population and sampling technique

Adults (aged ≥18 years) living with HIV and receiving ART at the MNH were consecutively enrolled during routine clinic visits. The participants provided written informed consent before data collection.

Inclusion criteria

Participants were eligible for inclusion if they had a confirmed diagnosis of HIV infection, were currently receiving ART for at least six months, and provided written informed consent to participate in the study.

Exclusion criteria

Participants were excluded if they had a known diagnosis of diabetes mellitus before ART initiation, were pregnant at the time of enrollment, lacked essential demographic or clinical information in their records, or were unable to provide informed consent.

Sample size determination

The sample size was calculated using the formula:

$n = \frac{z^2 \times p(1 - p)}{E^2}$

where n is the minimum sample size, z is the standard normal deviate corresponding to a 95% confidence level (1.96), p is the estimated population proportion (0.5), and E is the margin of error (0.05). After adjusting for a 10% non-response rate, the final required sample size was 427 participants. A total of 409 participants completed the study (95.8% response rate).

Data collection

Data were collected using a structured electronic questionnaire built into Research Electronic Data Capture (REDCap) software and administered on tablets [11,12]. The tool captured information on demographic characteristics (age, gender, education level, and employment status), lifestyle factors (including smoking and alcohol consumption), and clinical and laboratory parameters such as CD4 cell count, viral load, and body mass index (BMI). Additional data included the type and duration of the ART regimen, as well as family history of diabetes mellitus. Venous blood samples were collected from each participant after overnight fasting to determine the fasting plasma glucose levels.

Operational definitions

Diabetes mellitus was defined according to the WHO criteria as fasting plasma glucose ≥7.0 mmol/L or documented use of glucose-lowering medication. BMI was calculated as weight (kg) divided by height squared (m²) and was categorized as underweight (<18.5 kg/m²), normal (18.5-24.9 kg/m²), overweight (25-29.9 kg/m²), and obese (≥30 kg/m²). Viral-load suppression was defined as <1,000 copies/mL.

Statistical analysis

Data exported from REDCap were analyzed using SPSS version 26 (IBM Corp., Armonk, NY, USA). Continuous variables were summarized as mean ± standard deviation (SD) and range, and categorical variables as frequency (n) and percentage (%). Associations between independent variables and diabetes status were evaluated using logistic regression. Variables with a p-value <0.05 in the bivariate analysis were entered into a multivariate model to compute adjusted odds ratios (AORs) with 95% confidence intervals (CIs). A two-tailed p-value <0.05 was considered statistically significant.

## Results

Participant characteristics

A total of 409 participants were included in the analysis. Most participants were female (n = 267, 65.3%) and employed (n = 267, 65.3%). Educational attainment was generally high, with 25.9% (n = 106) having completed advanced secondary education and 39.4% (n = 161) holding university qualifications (Table 1).

**Table 1:** Sociodemographic and clinical characteristics of participants (N = 409). Categorical variables are presented as frequencies (n) and percentages (%). ART = antiretroviral therapy; BMI = body mass index; CD4 = cluster of differentiation 4

Variable	Category	N	%
Gender	Male	116	28.4
	Female	293	71.6
Employment status	Employed	267	65.3
	Unemployed	142	34.7
Education status	No education	7	1.7
	Completed primary school	49	12.0
	Completed Ordinary secondary school	86	21.0
	Completed Advanced secondary school	106	25.9
	Completed university	161	39.4
Do you smoke?	Yes	10	2.4
	No	399	97.6
Family history of diabetes	Yes	45	11.0
	No	364	89.0
Diabetes status	Yes (diabetic)	15	3.7
	No	394	96.3
CD4 <200 cells/µL	Yes	15	3.7
	No	394	96.3
BMI category	Normal	114	27.9
	Overweight	150	36.7
	Obese	145	35.5
Viral-load failure (≥1,000 copies/mL)	Yes	7	1.7
	No	402	98.3
ART line	First line	380	92.9
	Second line	29	7.1
ART regimen	Tenofovir disoproxil fumarate + lamivudine + dolutegravir (TDF + 3TC + DTG)	355	86.8
	Abacavir + lamivudine + dolutegravir (ABC + 3TC + DTG)	23	5.6
	Abacavir + lamivudine + atazanavir/ritonavir (ABC + 3TC + ATV/r)	9	2.2
	Tenofovir disoproxil fumarate + emtricitabine + atazanavir/ritonavir (TDF + FTC + ATV/r)	17	4.2
	Tenofovir disoproxil fumarate + emtricitabine + lopinavir/ritonavir (TDF + FTC + LPV/r)	2	0.5
	Dolutegravir + darunavir/ritonavir + etravirine (DTG + DRV/r + ETV)	1	0.2
	Zidovudine + lamivudine + atazanavir/ritonavir (AZT + 3TC + ATV/r)	1	0.2
	Tenofovir disoproxil fumarate + lamivudine + nevirapine (TDF + 3TC + NVP)	1	0.2

The mean age was 44.7 ± 14.4 years (range = 9.3-76.4). The mean BMI was 28.9 ± 9.4 kg/m² (Table 2), with 35.5% of participants classified as obese (Table 1).

**Table 2:** Descriptive statistics for continuous variables (N = 409). Continuous variables are presented as the mean ± standard deviation (SD). ART = antiretroviral therapy; BMI = body mass index; CD4 = cluster of differentiation 4

Variable	Mean ± SD	Range
Age (years)	44.7 ± 14.4	9.3–76.4
Duration on ART (years)	10.5 ± 4.5	0.1–26.5
Baseline CD4 (cells/µL)	742 ± 378	33–2,550
Body weight (kg)	68.1 ± 18.4	15.2–143.0
Height (m)	1.55 ± 0.20	0.60–1.95
BMI (kg/m²)	28.9 ± 9.4	12.2–86.9
Viral load (copies/mL)	1,310.5 ± 14,767.5	11–227,000

ART regimens and clinical profiles

**Figure 1:**
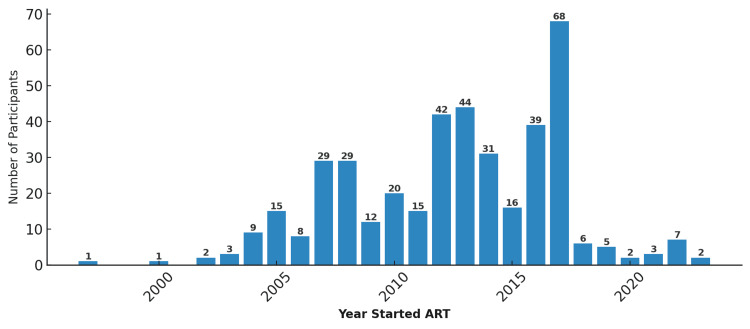
Number of participants initiating ART each year (1997–2023) at Muhimbili National Hospital, Tanzania (N = 409). The figure shows a rapid scale-up of ART uptake between 2010 and 2017, followed by a plateau in later years. ART = antiretroviral therapy

The predominant ART regimen was tenofovir disoproxil fumarate (TDF), lamivudine (3TC), and dolutegravir (DTG) (TDF + 3TC + DTG), used by 355 (86.8%) participants. A smaller proportion (7.1%) of patients received abacavir (ABC) + 3TC + DTG (ABC + 3TC + DTG). Most patients were receiving first-line therapy (92.9%), with only 7.1% receiving second-line regimens (Table 1). The mean duration of ART was 10.5 ± 4.5 years (range = 0.1-26.5), and the mean baseline CD4 count was 742 ± 378 cells/µL (Table 2). Most participants initiated treatment between 2012 and 2017 (Figure 1).

**Figure 2:**
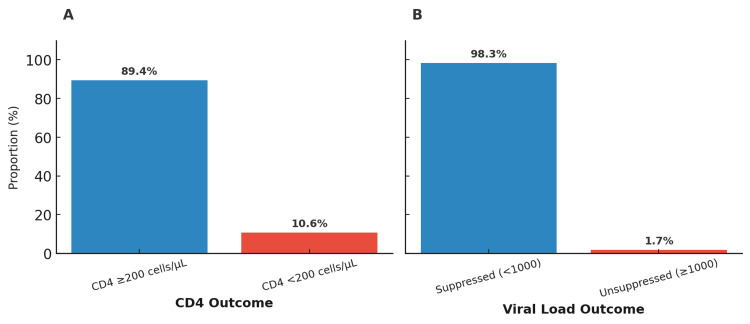
CD4 and viral load outcomes among participants on ART (N = 409). (A) Proportion of participants with CD4 count ≥200 cells/µL or <200 cells/µL. (B) Proportion of participants with suppressed (<1,000 copies/mL) or unsuppressed (≥1,000 copies/mL) viral loads. Both panels show favorable outcomes, with 89.4% of patients maintaining a CD4 count of 200 or higher and 98.3% achieving viral suppression. ART = antiretroviral therapy; CD4 = cluster of differentiation 4

Favorable treatment outcomes were achieved in the majority of participants. Figure 2A shows that 89.4% of the patients had CD4 ≥200 cells/µL, while Figure 2B shows that 98.3% achieved viral load suppression (<1,000 copies/mL).

Prevalence of diabetes mellitus and associated factors

**Figure 3:**
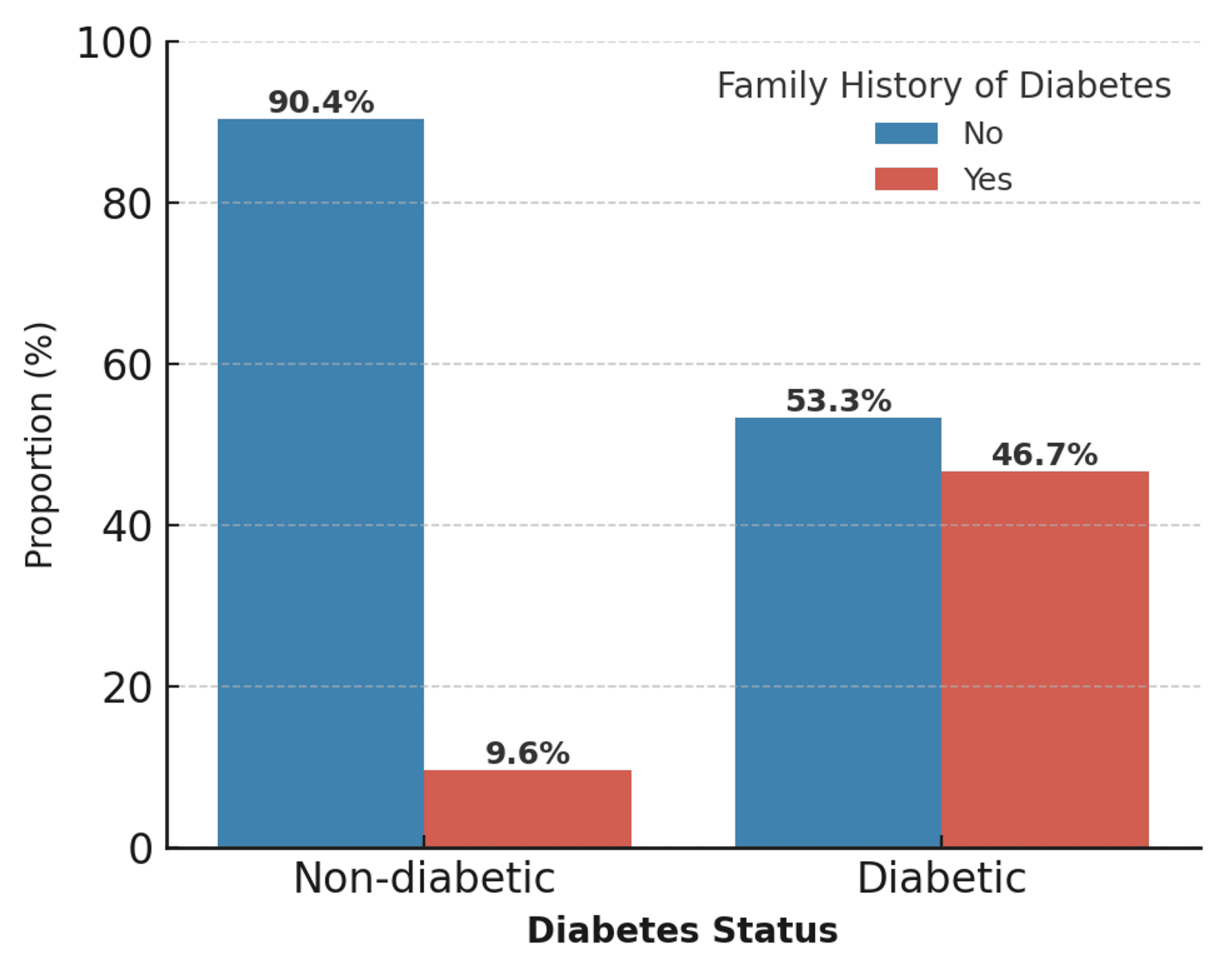
Association between family history of diabetes and diabetes status (N = 409). The proportion of participants with and without diabetes according to the presence of a family history of diabetes. This figure highlights the marked difference in the prevalence of diabetes between the two groups (χ² = 20.23, p < 0.001).

Overall, 3.7% (15/409) of the participants were diagnosed with diabetes mellitus. Diabetes was significantly associated with a family history of diabetes (χ² = 20.23, p < 0.001) (Figure 3).

In the univariate logistic regression, age, employment status, family history of diabetes, and CD4 count <200 cells/µL were significant predictors (Table 3).

**Table 3:** Univariate logistic regression of factors associated with diabetes among people living with HIV (N = 409). Note: Crude (unadjusted) ORs and 95% CIs for each variable in relation to diabetes status are shown. Variables with a p-value <0.05 were considered statistically significant and subsequently entered into the multivariate model. Significant variables (p < 0.05): age, employment status, family history of diabetes, and CD4 <200 cells/µL. ART = antiretroviral therapy; B = regression coefficient; S.E. = standard error; Wald = Wald chi-square statistic; CD4 = cluster of differentiation 4; BMI = body mass index; OR = odds ratio; CI = confidence interval

Variable	B	S.E.	Wald	P-value	Crude OR (Exp B)	95% CI for OR
Gender (male vs. female)	0.543	0.539	1.015	0.314	1.72	0.60–4.95
Age (years)	0.097	0.027	12.997	<0.001	1.10	1.05–1.16
Employment status (unemployed vs. employed)	1.080	0.538	4.031	0.045	2.94	1.03–8.45
Education status	–	–	2.588	0.629	—	—
Smoking (yes vs. no)	−1.117	1.089	1.053	0.305	0.33	0.04–2.76
Family history of diabetes (yes vs. no)	−2.104	0.545	14.903	<0.001	0.12	0.04–0.36
ART regimen	—	—	1.185	0.991	—	—
ART line (second vs. first)	0.069	1.054	0.004	0.948	1.07	0.14–8.44
Duration of ART (years)	−0.005	0.059	0.008	0.930	0.99	0.89–1.12
CD4 <200 cells/µL (yes vs. no)	−2.074	0.709	8.560	0.003	0.13	0.03–0.50
BMI (kg/m²)	−0.026	0.035	0.569	0.451	0.97	0.91–1.04
Viral-load failure (≥1,000 vs. <1,000 copies/mL)	−1.515	1.114	1.849	0.174	0.22	0.03–1.95

In the multivariate logistic regression model, older age (AOR = 1.14; 95% CI = 1.05-1.25; p = 0.003) and absence of a family history of diabetes (AOR = 0.07; 95% CI = 0.02-0.27; p < 0.001) remained significant independent predictors of diabetes, while having a CD4 count <200 cells/µL also showed borderline significance (AOR = 0.14; 95% CI = 0.02-0.88; p = 0.036) (Table 4).

**Table 4:** Multivariate logistic regression identifying independent predictors of diabetes among people living with HIV (N = 409). AORs with 95% CIs from the final model, including age, employment status, family history of diabetes, CD4 count <200 cells/µL, and viral-load failure, are shown. Significant predictors (p < 0.05) are in bold. CD4 = cluster of differentiation 4; AOR = adjusted odds ratio; CI = confidence interval

Variable	B	S.E.	Wald	P-value	AOR (Exp B)	95% CI for AOR
Viral-load failure (≥1,000 copies/mL)	−2.183	1.857	1.382	0.240	0.11	0.00–4.29
Age (years)	0.133	0.044	9.099	0.003	1.14	1.05–1.25
Employment status (unemployed vs. employed)	0.014	0.827	<0.001	0.986	1.01	0.20–5.13
Family history of diabetes (yes vs. no)	−2.637	0.674	15.308	<0.001	0.07	0.02–0.27
CD4 < 200 cells/µL (yes vs. no)	−1.938	0.922	4.418	0.036	0.14	0.02–0.88
Constant	−4.454	2.624	2.881	0.090	0.012	—

## Discussion

In this study, 427 PLWHIV were recruited, of whom 409 (95.8%) consented to participate. The mean age was 44.7 years, representing a mature cohort with prolonged exposure to HIV infection and ART. Multivariate logistic regression identified older age and low CD4 count (<200 cells/µL) as significant risk factors for diabetes mellitus, whereas the absence of a family history of diabetes was protective. These findings align with those of earlier studies, while differing from others, underscoring the multifactorial nature of diabetes risk in this population [5-8,13].

Although this study did not find independent associations between absolute CD4 count, viral-load failure, or BMI and diabetes mellitus risk, a study conducted at the MNH reported these variables as significant predictors [8]. Such differences may be due to variations in participant characteristics, healthcare access, or clinical management practices. In contrast, studies from northern Ethiopia and Taiwan identified other predictors, including hypertriglyceridemia and hypertension, which were not included in our analysis [5,13].

Contrary to our results, another study from northern Ethiopia found that prolonged ART duration was a major risk factor for diabetes [13]. However, consistent with our research, old age was a significant predictor of diabetes mellitus. Similarly, a study from Botswana highlighted efavirenz-based regimens as substantial risk factors, an association absent in our cohort, again reflecting regimen diversity across settings [6].

The strong association between family history and diabetes mellitus observed in this study is consistent with broader epidemiological evidence. The EPIC-InterAct Project demonstrated that a positive family history is a powerful, independent predictor of type 2 diabetes, even after adjusting for lifestyle, anthropometric, and genetic factors [14]. These findings highlight the persistent influence of hereditary predisposition on diabetes risk in PLWHIV and emphasize the importance of routinely documenting family history in clinical assessment and risk stratification.

Collectively, these findings and previous reports underscore the importance of integrating NCD care into HIV treatment programs. A study from KwaZulu-Natal, South Africa, reported a high prevalence of diabetes among PLWHIV, particularly among women, many of whom had poor glycemic control [7]. This evidence underscores the importance of routine diabetes screening and enhanced metabolic monitoring in ART clinics to prevent complications and improve patient outcomes.

Our observations also align with earlier Tanzanian research, which found that lower CD4 counts and high BMI are associated with an increased risk of diabetes. However, this association was not statistically significant in our dataset [8]. Variations in ART duration, adherence, or body composition assessment methods may partly explain these differences. Moreover, some studies have reported an underdiagnosis of diabetes due to reliance on fasting glucose alone [15], suggesting that the prevalence in our study could be underestimated.

Integrated care models that combine HIV and NCD management may improve resource utilization and clinical outcomes. Evidence from Zimbabwe indicates that implementing electronic data systems across HIV, tuberculosis, and NCD programs strengthens case detection and care coordination [2]. Future studies should adopt longitudinal or cohort designs to better characterize the progression of metabolic abnormalities and their interaction with ART [4,9,10].

Our findings reinforce the growing consensus that HIV programs must extend beyond achieving viral suppression to address chronic metabolic complications among PLWHIV [4,10]. Incorporating health education on nutrition, physical activity, smoking cessation, alcohol moderation, and weight control into ART services is essential for reducing the risk of diabetes and improving long-term health outcomes [9,15]. Evidence from South Africa and Zimbabwe supports this integrated model as a practical and cost-effective approach in resource-limited settings [2,4,7,9,10]. By adopting such strategies, health systems can better manage both infectious and chronic diseases, thereby enhancing the quality of life and survival of PLWHIV [4,9].

Study limitations

This cross-sectional study design limits the ability to make causal inferences between the identified risk factors and diabetes. Conducted at a single tertiary center (MNH), the findings may not represent the broader Tanzanian PLWHIV population. Self-reported lifestyle data may introduce recall bias, and the lack of long-term follow-up precludes the evaluation of risk evolution over time. Despite statistical adjustments, residual confounding factors may persist. Additionally, BMI does not fully capture body fat distribution, and reliance on fasting plasma glucose alone may lead to an underestimation of diabetes prevalence.

## Conclusions

This study highlights the importance of routine blood glucose monitoring among PLWHIV receiving ART. Although ART markedly improves survival and immune recovery, diabetes mellitus was observed in 3.7% of this clinic cohort, with higher absolute counts among older adults and those with lower CD4 levels. In contrast, the absence of a family history of diabetes appears to be protective. Integrating regular metabolic screening, nutritional counseling, and targeted health education into HIV treatment programs can enable early detection and timely intervention. Embedding diabetes prevention and management within existing HIV care frameworks will help sustain the long-term benefits of ART while addressing metabolic health within routine service delivery. However, the excess diabetes risk relative to the general Tanzanian population cannot be inferred from these findings, as no age-standardized comparison group was included and diagnostic ascertainment was limited to fasting plasma glucose rather than glycated hemoglobin or an oral glucose tolerance test. As this was a prevalence study, we did not assess causality or the contribution of specific determinants such as ART exposure patterns, stress, diet, or other metabolic factors. Future analytical studies (e.g., case-control or longitudinal cohort designs) are needed to determine the extent to which HIV infection, ART, and individual-level risk factors contribute to diabetes development in this population.
